# Gender Differences in Mental Health Disorder and Substance Abuse of Chinese International College Students During the COVID-19 Pandemic

**DOI:** 10.3389/fpsyt.2021.710878

**Published:** 2021-08-17

**Authors:** Mingsheng Li, Heng Su, Zhengluan Liao, Yaju Qiu, Yan Chen, Junpeng Zhu, Yangliu Pei, Piaopiao Jin, Jiaxi Xu, Chang Qi

**Affiliations:** ^1^Department of Psychiatry, Zhejiang Provincial People's Hospital, Zhejiang, China; ^2^Psychology Department, Denison University, Granville, OH, United States; ^3^Graduated School, BengBu Medical College, Bengbu, China; ^4^The Second Clinical Medical College, Zhejiang Chinese Medical University, Hangzhou, China

**Keywords:** mental health, substance use disorder, college students, COVID-19, gender difference

## Abstract

**Introduction:** The outbreak of coronavirus disease has negatively impacted college students' mental health across the world. In addition, substance abuse also is trouble among these students. This study aims to find the gender difference in Chinese international college students' mental health and substance abuse during the COVID-19 pandemic.

**Method:** We conducted an online survey using PHQ-9, GAD-7, and several questions related to substance abuse frequency, self-injury, and suicidal thoughts, 535 male and 475 female Chinese international college students whose ages ranged from 18 to 23 years old (**x** = 20.19, SD = 1.50) were recruited during the epidemic. We utilized *t*-test and binary logistic regression in our study to find out the difference and statistical significance between substance abuse issues and mental health problems across gender.

**Results:** Both male and female Chinese international college students had statistical significance with self-injury ideas and behaviors (*t* = −2.21, *p* < 0.05). Furthermore, the male college students with anxiety problems had positive statistical significance with medicine (*OR* = 3.47, *95%CI* = 1.45–8.30, *p* < 0.01) and negative statistical significance with drinks (*OR* = 0.23, 95%CI = 0.08–0.65, *p* < 0.01). While for female college students with an anxiety problem, they had positive statistical significance with medicine (*OR* = 4.88, *95%CI* = 1.53–15.57, *p* < 0.01), drugs (*OR* = 4.48, *95%CI* = 1.41–14.25, *p* < 0.05) and cigarettes (*OR* = 6.63, *95%CI* = 1.95–22.57, *p* < 0.01) and negative statistical significance with drinks (*OR* = 0.18, *95%CI* = −0.05 to 0.65, *p* < 0.01).

**Conclusion:** This is the first cross-sectional study focusing on the Chinese international college students' mental health and substance abuse problems during the COVID-19 pandemic. We found that Chinese international college students' mental health and substance abuse situation has been negatively influenced during this period. In addition, the self-injury ideas and behaviors also showed a high tendency for these students. The findings of our study also highlight the need to find more interventions and preventions to solve the different mental health and substance abuse problems for college students, especially for female Chinese international college students.

## Introduction

Since the beginning of 2020, the rapid outbreak of the novel coronavirus disease (COVID-19) has been threatening the health of human beings across the world. As a public health emergency, it generates a series of psychological consequences, from mental health problems and substance abuse disorders to behavioral changes (1, 2). International college students, who come from across the world with different cultural backgrounds, are one of the populations that have been affected significantly.

The amalgamation of discontinuing University studies due to the COVID-19 outbreak and the lasting change in the academic environment that shifts to take the classes remotely, and the emergence of feelings of fear and anxiety, have all occurred with this student's population (3, 4). Furthermore, new sources of stress have been created and intensified, which include the long period of separation from the outside world, health concerns over the public themselves or the people around them, excessive fears of being infected, and stigma. These young college students are easily overwhelmed by the inaccurate information, published and posted online and on social media, which increased their anxiety and fear (5–8). In addition, gender differences in mental health and substance abuse are urgent for psychosocial and medical concerns, especially for the Chinese college international students during the COVID-19 pandemic.

Most clinical and epidemiological studies on depression disorder found out higher prevalence rates among females than males, who have twice the lifetime rates of depression of the latter. The World Health Organization (9) predicted that depression would become one of the severe diseases by 2030 for females worldwide. In 2010, the global annual prevalence rates of depression of female students and male students were 5.5 and 3.2%, respectively (10–12). For anxiety disorders, various researches showed that they are more prevalent among females than males. Females have a higher tendency to develop such disorders, with the lifetime and past-year rates of anxiety disorders being 1.5–2 times higher than males (13, 14). Many previous studies also pointed out that female college students are facing more severe mental health issues that they are at increased risk of loneliness, depression, anxiety, and high self-injury tendency during COVID-19 pandemic, which should be an urgent issue that needs to be solved (15–17).

Similar results could also be obtained for substance abuse among college students, such as medicine and drugs (18). For example, some studies indicated that female college students tend to use heroin more rapidly, thus becoming addicted to it in a shorter period compared to male students (19). In addition, previous studies indicated that college students were at high risk for poor sleep quality during the COVID-19 so that frequently taking medicine such as sleep pills and antalgic is a common phenomenon among these students (20–22). Previous studies also identified both female and male substance abusers that are usually diagnosed with emotional disorders. For example, studies on opiate and cocaine abusers discovered higher percentages affective and anxiety disorders in female students than in male students (23).

In light of growing concerns associated with the influence of COVID-19 on the mental health of Chinese international college students, there is an urgent need for research to address the mental health burden of these college students across genders. This study first analyzed prevalence in mental health by taking a sample of Chinese international college students during the COVID-19 pandemic. It then explores the difference between mental health and substance abuse problems in genders, so as to determine whether there is a gender difference and to analyze specific issues. Finally, the findings of our study give evidence for solving the mental health and substance abuse problems of college students.

## Materials and Methods

### Participants

This study was designed as an online and anonymous questionnaire implemented from December 31, 2020, to January 9, 2021, 1 month after most Chinese international students finished their fall semester courses. The online survey was developed on the official website of “Questionnaire Star,” which is recognized as a professional online questionnaire and evaluation platform, 1,045 participants were recruited from the Chinese college student population who were studying in the United States. This study utilized 1,010 participants after excluding graduate-degree students. Gender distribution among the participants was 535 males and 475 females, whose ages ranged from 18 to 23 years old ($\bar{x}$ = 20.19, *SD* = 1.50).

### Measurements

The series of questions involved in this study are mainly composed of six sections, and the specific items and scores are shown in tables and Appendixes, including the demographic questions, PHQ-9, GAD-7, and questions related to the frequency of substance abuse, self-injury, and suicidal thoughts. According to the score criteria of each questionnaire, this study will explore whether the students are experiencing mental health disorders. For frequency of the substance, our team supposed that the participants with abuse problems were at least taking certain substances above three times a week. We utilized the *t*-test and binary logistic regression analysis in our study to find out the difference between substance abuse issues and mental health problems across different genders in Chinese international college students. A two-tailed *p*-value of <0.05 was considered significant for all tests. We used *Cox and Snell R*^2^ and *Nagelkerke R*^2^ to evaluate our model in that the larger the value of *R*^2^, the better a model's *Goodness of Fit* is. In our model of binary logistics regression of different genders, both male and female groups showed a good *Goodness of Fit*.

## Results

Table 1 indicates the basic demographic characteristics, depression levels, and anxiety levels of the student groups under different genders. Although both gender groups do not have statistical significance with overall depression and anxiety scores, these groups had statistical significance with self-injury ideas and behaviors (*t* = −2.21, *p* < 0.05). Table 2 shows the frequency of the substance abuse per week for Chinese international college students. The result showed these students had statistical significance with drugs (*t* = 2.213, *p* < 0.05) and cigarettes (*t* = 2.602, *p* < 0.01).

**Table 1:** Descriptive information of male and female Chinese international college students (*n* = 1,010).

**Characteristics**	**Gender**				***t***	***p***
	**Male (** ***n*** **= 535)**		**Female (** ***n*** **= 475)**			
	***n***	**%**	***n***	**%**		
**Age**					−1.18	0.24
18–20	350	65.4	282	59.4		
21–23	185	34.6	193	40.6		
**Class year**					−0.37	0.71
Freshman	110	20.6	112	23.6		
Sophomore	203	37.9	147	30.9		
Junior	136	25.4	133	28.0		
Senior	86	16.1	83	17.5		
**Suicidal thoughts and behavior**						
Self-injury ideation	125	23.4	137	28.6	−1.98	**<0.05**
Suicidal attempt	96	17.9	99	20.7	−1.16	
**PHQ-9**					1.23	0.22
**GAD-7**					−0.88	0.38

*The bold values mean these results are statistically significant*.

**Table 2:** Frequency of substance abuse per week of male and female Chinese international college students (*n* = 1,010).

**Characteristics**	**Gender**				***t***	***p***
	**Male (** ***n*** **= 535)**		**Female (** ***n*** **= 475)**			
	***n***	**%**	***n***	**%**		
**Alcohol**					0.952	0.34
0 times	230	43.0	214	45.1		
1–3 times	265	49.5	233	49.1		
4–6 times	40	7.5	28	5.9		
>7 times	/	/	/	/		
**Medicine**					1.938	0.05
0 times	231	43.2	234	49.3		
1–3 times	223	41.7	185	38.9		
4–6 times	57	10.7	35	7.4		
>7 times	24	4.5	21	4.4		
**Desserts**					−1.116	0.27
0 times	192	35.9	154	32.4		
1–3 times	270	50.5	246	51.8		
4–6 times	25	4.7	30	6.3		
>7 times	48	9.0	45	9.5		
**Drinks**					−1.449	0.15
0 times	229	42.8	182	38.3		
1–3 times	213	39.8	217	45.7		
4–6 times	47	8.8	43	9.1		
>7 times	46	8.6	33	6.9		
**Drugs**					2.213	**<0.05**
0 times	222	41.5	230	48.4		
1–3 times	225	42.1	171	36.0		
4–6 times	46	8.6	37	7.8		
>7 times	42	7.9	37	7.8		
**Cigarette**					2.602	**<0.01**
0 times	230	43.0	243	51.2		
1–3 times	240	44.9	178	37.5		
4–6 times	30	5.6	19	4.0		
>7 times	35	6.5	35	7.4		

*The bold values mean these results are statistically significant*.

**Figure 1:**
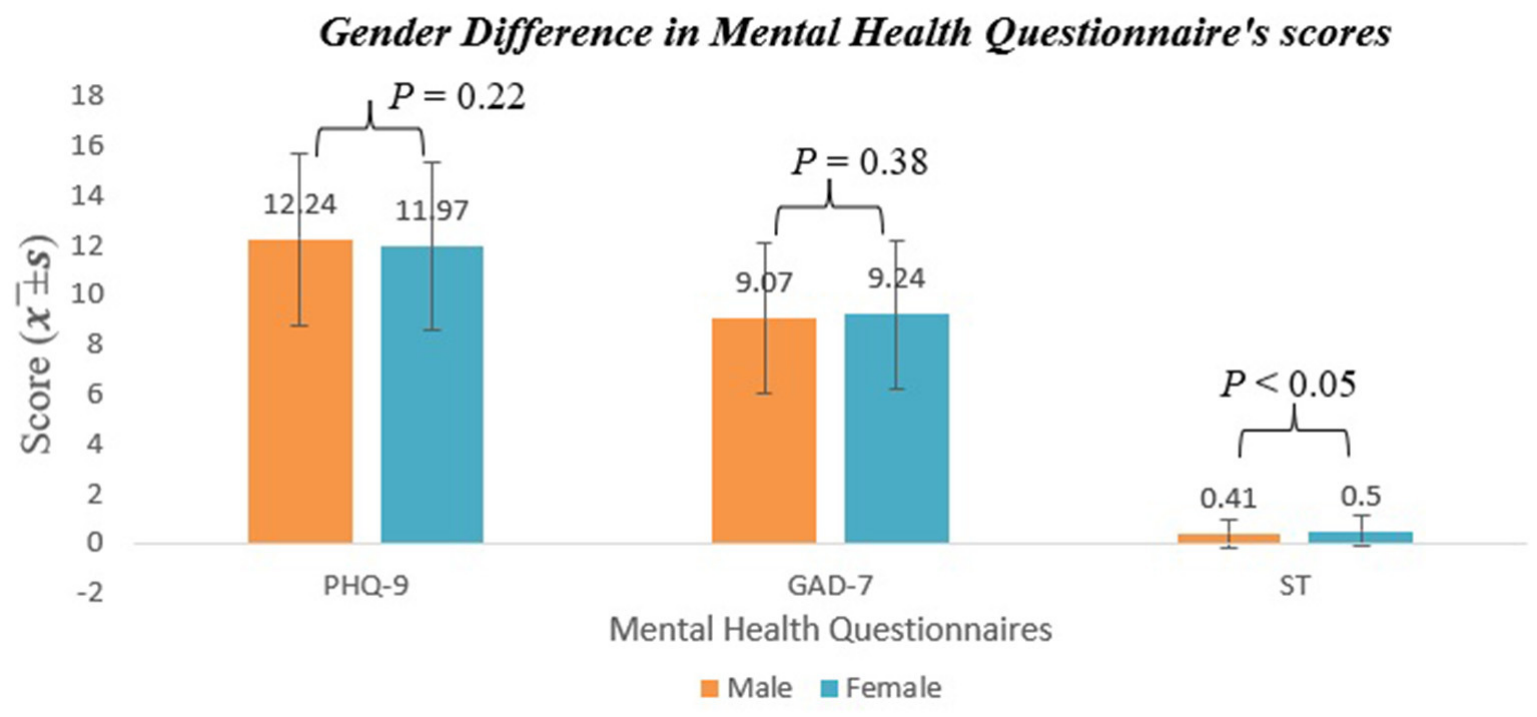
Gender difference in mental health questionnarie's score.

Figure 1 reveals the average score and standard deviation ($\bar{x}\pm s$) for three mental health questionnaire scores and the frequency of substance abuse situations on gender difference, respectively. Comparing the PHQ-9 scores of female and male Chinese international college students shows that males have a higher $\bar{x}\pm s$ (12.24 ± 3.48) than females (11.97 ± 3.40), which means that males were experiencing the low mood more severely than females. In contrast to these two questionnaires, females get a $\bar{x}\pm s$ on the GAD-7 (9.24 ± 2.98) and ST (0.50 ± 0.62), indicating that they are more likely to be subject to anxiety disorder and suicidal behavior. For substance frequency, the female students show a higher $\bar{x}\pm s$ on desserts (1.16 ± 0.37) and snacks (1.22 ± 0.41), while males have high $\bar{x}\pm s$ on alcohol (1.07 ± 0.26), medicine (1.15 ± 0.36), drinks (1.17 ± 0.38), and cigarettes (1.12 ± 0.33). This result means the male Chinese international college students more easily drink alcohol and beverages, take medicine, and smoke. However, the female students are more addicted to desserts and snacks.

Table 3 indicates the difference in gender with anxiety problems and substance problems for male and female Chinese international college students. The male college students with anxiety problems had positive statistical significance with medicine (*OR* = 3.47, *95%CI* = 1.45–8.30, *p* < 0.01) and negative statistical significance with drinks (*OR* = 0.23, *95%CI* = 0.08–0.65, *p* < 0.01). This result shows the probability of taking medicine by male college students with anxiety problems is 3.473 times higher than that of male students without anxiety problems. Meanwhile, the probability of drinking beverages by male students with anxiety problems is 0.23 times higher than that of male students without anxiety problems.

**Table 3:** Binary logistic regression analysis for anxiety disorder and substance abuse.

	**Males**							**Females**						
	**B**	**SE**	**β**	**p**	**OR**	**95%** ***CI***		**B**	**SE**	**β**	**p**	**OR**	**95%** ***CI***	
						**Lower**	**Upper**						**Lower**	**Upper**
Alcohol	−0.29	0.44	0.14	0.51	0.75	0.32	1.77	0.55	0.51	1.18	0.28	1.73	0.64	4.67
Medicine	1.25	0.45	5.46	<0.01**	3.47	1.45	8.30	1.59	0.59	7.17	<0.01**	4.88	1.53	15.57
Dessert	−0.48	0.53	0.61	0.36	0.62	0.22	1.73	0.91	0.54	2.82	0.093	2.46	0.86	7.12
Drinks	−1.46	0.52	23.64	<0.01**	0.23	0.08	0.65	−1.72	0.66	6.82	<0.01**	0.18	0.05	0.65
Drugs	0.55	0.44	0.05	0.21	1.73	0.73	4.08	1.50	0.59	6.43	<0.05*	4.48	1.41	14.25
Cigarette	0.62	0.43	1.35	0.15	1.86	0.80	4.32	1.89	0.63	9.17	<0.01**	6.63	1.95	22.57
Snacks	0.58	0.42	7.86	0.17	1.78	0.78	4.07	−0.39	0.57	0.49	0.49	0.67	0.22	2.04

Significant P < 0.05* and < 0.01**. The bold values mean these results are statistically significant.

For female college students with anxiety problem, they had positive statistical significance with medicine (*OR* = 4.88, *95%CI* = 1.53–15.57, *p* < 0.01), drugs (*OR* = 4.48, *95%CI* = 1.41–14.25, *p* < 0.05), and cigarettes (*OR* = 6.63, *95%CI* = 1.95–22.57, *p* < 0.01). These female students also had negative statistical significance with drinks (*OR* = 0.18, *95%CI* = −0.05 to 0.65, *p* < 0.01). This result shows that the probability of taking medicine, drugs, and cigarettes by female Chinese international college students with anxiety problems is 4.88, 4.48, and 6.63 times higher than that of female students without anxiety problems. Meanwhile, the probability of drinking beverages by male students with anxiety problems is 0.18 times higher than that of students without anxiety problems. However, we do not find any statistical significance for both gender groups under depression problems.

## Discussion

This cross-sectional study is the first study focusing on the Chinese international college students' mental health and substance abuse problems during the outbreak of COVID-19. There were two main findings in this study—the prevalence of mood problems and substance abuse in Chinese international college students and the difference between these two issues across gender. First, both male and female Chinese international college students showed a high prevalence rate of depression and anxiety problems. Secondly, both male and female Chinese international college students with anxiety problems showed a higher tendency to take medicines and drink beverages. Besides, female Chinese international college students are more vulnerable to experiencing anxiety problems with drugs and cigarettes.

The reason causing these students to have a higher tendency to suffer poor mental health might be attributed to the disruption of their academic routines. Many universities were transitioning to remote learning after the spring break in 2020 that these students could not adapt to the unfamiliar teaching style quickly. Most of these international students living on campus were urged to leave their residence halls in a few days by the school, which led them to not go back to campus for a long time and cease their research projects, work, and internships. Chinese international college students were not only facing stress from academics but also experiencing distress from social and daily life. For instance, they need to overcome such problems as time differences to stay up late to take classes remotely, concerns for health, social isolation, VISA issues, and limited and expensive flights (24). Moreover, the COVID-19 pandemic drove those international college students to delay their graduation, or they even cannot attend graduation in person, which raises anxiety levels. Recently, America's *ICE* and the Student and Exchange Visitor Program (25) have jointly published the newest statistical report on international students, *SEVIS By the Numbers*, which showcases the specific numbers and countries of international students influenced by the pandemic in this community. Although 1,251,569 students activated F-1 and M-1 status in 2020, the number decreased by 17.86 percent compared to 2019. Thus, the number of Chinese students attending American colleges is decreasing, caused by the concerns of COVID-19 and a series of prohibitions. The stress level among Chinese international college students is drastically increasing by these prohibitions and policies, especially for female students. The female students may be overwhelmed by the outbreak of the COVID-19 and could not adapt to the new life not only in an academic way but also in their daily life. As a result, they may concerned by more factors than the male students, thereby increasing their stress levels. These female Chinese international college students should have a conversation with their parents and a therapist to express their thoughts and let these people help them.

The self-injury ideas and behavior uncover a notable difference between female and male Chinese international college students in our study in that 25.94% of students reported self-injury ideas, especially in the female students. Previous studies on self-injury and suicidal thoughts among college students reported that one in five students has had thoughts of suicide, including 9% attempted suicides and around 20% reported self-injuries, based on more than 67,000 college students (26, 27). The immaturity of psychological development of college students caused them to self-injure and have suicidal attempts. The female college students with depression disorder have a higher tendency of committing self-injury behavior (28, 29). Similar results have been reported: females are more likely to commit self-injury and individuals who have been socially isolated or who stay at home show some negative feelings or thoughts of self-harm and suicide (30–32). Therefore, their professors, friends, and family members should prompt awareness regarding suicidal thoughts and attempts so as to reduce self-injury rates, particularly for female Chinese international college students. These female students are a vulnerable group shown in our study, and once finding these people attempt to do suicidal and self-injury behaviors, the people around these female students, should immediately send them to see therapists and doctors.

Besides, many studies on substance abuse indicate the gender significance in substance-related epidemiology, social factors, and psychiatric disorders (33). College students are usually associated with poor sleep, to be more specific, they have sleep difficulties that they are constantly forced to stay up late by academic stress and assignment workload (34), or they get up very late due to social factors (parties and hang out with friends). Before the epidemic, the nightlife of college students is full of fun and engaging that they forget about the time (35, 36). In order to get up early for the second day's class, they need to sleep as soon as possible. Sleep medicine would be an effective tool they could use. For academic stress, these college students almost need to take sleep medicine every weekday. Whether male or female, they are under tremendous stress from the assignments and exams. At the same time, they also need to stay up late to take the class since most Chinese international college students go back to China and take classes remotely. Therefore, they need to stay up at 3:00 a.m. or even later for taking the class (37–39). Their physical and psychological health collapsed under the pressure of stress from academic life, social activities, and internship, and this explains why they would get addicted to sleep medicines to have a good sleep. For both male and female students, they should find a new sleep schedule for themselves so that they could have enough high-quality sleep to keep the balance for their health.

Another explanation for solving stress from academic and daily life is drinking some sweet drinks, such as bubble tea that is the trend across the world, especially in China. The young Chinese generations, the college students, are the major consumers of bubble tea. A prior study reported that college students would order bubble tea at least four times a week. Except for water, bubble tea is the second liquid they drink frequently, some people even reported using bubble tea to replace water. Sugar is one of the ingredients to make bubble tea, making college students release stress and anxiety from their daily work and study (40–42). Thus, it might be the reason causing why these Chinese college students, especially female students, frequently drink beverages. Similar results can be found in the previous study that females are more prone to eating and getting addicted to sweet food (43). The learning process at midnight drastically increased stress in Chinese international college students during the pandemic. Therefore, they need to eat some sweets to improve their mood and release stress. We hope these students could find a more effective way to overcome anxiety, like running, reading, and doing something they like instead of eating too many sweet foods.

In addition to these two substances, the female Chinese international college students experiencing anxiety also had statistical significance with drugs and cigarettes. Drug abuse is a problematic issue in college students across the world that deserves much attention (44–47). A study focused on gender differences in cocaine abuse indicated that females addicted to opioids showed a high tendency of taking heroin (48). Another study also finds that cocaine use is more frequent in females than in males (49, 50). The students who take drugs might believe the drugs could help them get rid of sad emotions and do not fall into annoyance easily. They want to escape from reality, so as not to become worried about academic life and daily life. However, drugs are illegal in China, some states in America, and many countries, and the governments should supervise them more strictly. Female Chinese international college students should go to the Addiction Treatment Center to do drug rehabilitation. They could also talk to the family members and professors about their issues so that these people could help them to solve the problems.

For the cigarette abuse problem in the Chinese international college student population. According to the Gender and Mental Health (2015) study by the WHO, males are more likely to be addicted to cigarettes than females, but our study finds an opposite result. Maybe because the males did not count how many cigarettes they smoked every week, the frequency they provided is an approximate number in our study. There is also a possibility for females to smoke because the price of cigarettes is low in China. In a word, they can smoke anywhere and anytime, not like alcohol and drugs, which need a particular place and occasion to take. In America, the American Lung Association has advocated for the age of sale for tobacco products from 18 to 21 (51), so it is difficult and illegal for people to get tobacco. Furthermore, epidemiologic studies have found strong associations between nicotine dependence and mood and anxiety disorders, and smoke is a common way to cope with anxiety problems for individuals during the pandemic (52–55). Therefore, Chinese international college students need to decrease the frequency of smoking, both males and females. In addition, they need to find replacement methods for smoking, like chewing gum and taking a deep breath when they crave tobacco.

It can be said that substance abuse had a significant effect on both male and female Chinese international college students as regards anxiety disorder. COVID-19 significantly enhances the suicidal and self-injury tendency among Chinese international college students, who have already been put under tremendous academic, social, and daily life pressure, especially female Chinese international college students.

This study has several limitations that should improve in future researches. First, its sample size is relatively small. A large number of questions in the survey caused students to be unwilling to spend time to participate and complete it. Besides, the authority of participants' actual mental health situation still needs to be evaluated by the psychiatrists and clinical psychologists since they completed the questionnaires without the supervision of our team members.

The second issue is that the participant may not tell us the truth, like the deep secrets (self-injury and suicidal ideas) in their mind, although we informed them that the survey is confidential and anonymous. In addition, the participants might forget their situation since we asked them to complete the survey when they had already finished their semester for a month. For these limitations, it is necessary to shorten the length of items and use other methods for analysis. For example, we could conduct a longitudinal study that collects the data for mental health change in participants after 3 months to find any changes and differences for them. We could also collect the data from domestic American students to compare with Chinese international students to find the difference.

## Conclusion

Our study is a cross-sectional study investigating the prevalence of mental health problems and substance abuse among Chinese international college students during the COVID-19 pandemic. We revealed a critical concern, the female Chinese international college students encountering more severe problems than male students, whether in terms of mental health problems or substance abuse. The students who deliver higher scores in each questionnaire should go to the psychiatry department to be evaluated by the psychiatrists and therapists to uncover their deep thoughts and problems. College counselor service faculty need to develop effective ways to support these students. The parents of these college students should be taking care of them more to alleviate the their mental health burden and make them feel they are being loved. Their self-injury ideas and behaviors also need to be a concern.

## Data Availability Statement

The original contributions presented in the study are included in the article/supplementary material, further inquiries can be directed to the corresponding author/s.

## Ethics Statement

The studies involving human participants were reviewed and approved by Zhejiang Provincial People's Hospital Denison University. The patients/participants provided their written informed consent to participate in this study.

## Author Contributions

ML and CQ conceived the study, did the literature review, and drafted the report. CQ and ML did statistical analyses. ML, CQ, HS, ZL, YQ, JZ, YC, YP, PJ, and JX collected the data. ML took the lead in writing the manuscript. CQ, HS, ZL, YQ, JZ, YC, YP, PJ, and JX commented on the manuscript. All authors contributed to the article and approved the submitted version.

## Conflict of Interest

The authors declare that the research was conducted in the absence of any commercial or financial relationships that could be construed as a potential conflict of interest.

## Publisher's Note

All claims expressed in this article are solely those of the authors and do not necessarily represent those of their affiliated organizations, or those of the publisher, the editors and the reviewers. Any product that may be evaluated in this article, or claim that may be made by its manufacturer, is not guaranteed or endorsed by the publisher.

## Appendix

**TABLE A1:** Gender difference in PHQ-9 item scores ($\bar{x}\pm s$).

**PHQ-9**	**Male (*n* = 535)**	**Female (*n* = 475)**	***t***	***p***
1. Little interest or pleasure in doing things?	1.36 ± 0.99	1.39 ± 0.98	0.405	0.69
2. Feeling down, depressed, or hopeless?	1.02 ± 0.99	1.34 ± 0.97	0.929	0.35
3. Trouble falling or staying asleep, or sleeping too much?	1.38 ± 1.00	1.45 ± 1.03	–1.079	0.28
4. Feeling tired or having little energy?	1.38 ± 0.98	1.34 ± 1.02	0.642	0.52
5. Poor appetite or overeating?	1.29 ± 1.00	1.37 ± 1.01	–1.246	0.21
6. Feeling bad about yourself—or that you are a failure or have let yourself or your family down?	1.39 ± 1.02	1.36 ± 1.00	0.426	0.67
7. Trouble concentrating on things, such as reading the newspaper or watching television?	1.37 ± 1.00	1.20 ± 0.99	2.663	**<0.01**
8. Moving or speaking so slowly that other people could have noticed. Or the opposite—being so fidgety or restless that you have been moving around a lot more than usual?	1.36 ± 1.00	1.30 ± 1.00	0.919	0.36
9. Thoughts that you would be better off dead, or thoughts of hurting yourself in some way?	1.30 ± 1.04	1.26 ± 1.00	0.620	0.54
Overall score	12.24 ± 3.48	11.97 ± 3.40	1.229	0.22

*PHQ-9: Patient Health Questionnaire-9. **Scores ≤4** suggest minimal depression. **Scores 5–9** suggest mild depression. **Scores 10–14** suggest moderate depression severity. **Scores 15–19** suggest moderately severe depression. **Scores 20 and greater** suggest severe depression. The bold values mean these results are statistically significant*.

**TABLE A2:** Gender difference in GAD-7 item scores ($\bar{x}\pm s$).

**GAD-7**	**Male (*n* = 535)**	**Female (*n* = 475)**	***t***	***p***
1. Feeling nervous, anxious, or on edge?	1.36 ± 0.99	1.39 ± 0.98	–0.556	0.58
2. Being unable to stop or control worrying?	1.33 ± 1.02	1.34 ± 0.99	–0.065	0.95
3. Worrying too much about different things?	1.37 ± 1.02	1.35 ± 1.02	0.197	0.84
4. Having trouble relaxing?	1.22 ± 1.01	1.26 ± 0.98	–0.551	0.58
5. Being so restless that it is hard to sit still?	1.32 ± 0.98	1.35 ± 1.00	–0.572	0.57
6. Becoming easily annoyed or irritable?	1.20 ± 0.98	1.27 ± 0.98	–1.121	0.26
7. Feeling afraid, as if something awful might happen?	1.28 ± 0.99	1.28 ± 1.00	–0.020	0.98
Overall score	9.07 ± 3.03	9.24 ± 2.98	–0.881	0.38

*GAD-9: General Anxiety Disorder-7. GAD-7 total score for the seven items ranges from 0 to 21. **Scores of 5, 10, and 15** represent cut-points for mild, moderate, and severe anxiety, respectively*.

**TABLE A3:** Gender difference in ST item scores ($\bar{x}\pm s$).

**Suicidal tendency**	**Male (*n* = 535)**	**Female (*n* = 475)**	***t***	***p***
1. Have you ever thought or did self-injury in the past year? (Behaviors such as pulling hair, cutting wrists/arms, scratching, and fingernail biting, etc.)	0.23 ± 0.42	0.29 ± 0.45	–1.984	**<0.05**
2. Have you ever thought or did suicide behave in the past year?	0.18 ± 0.38	0.21 ± 0.41	–1.164	0.25
Overall score	0.41 ± 0.58	0.50 ± 0.62	–2.216	**<0.05**

*The “1” presents “Yes” and “0” presents “No” for each question on substance use. The bold values mean these results are statistically significant*.
